# Celiac Trunk Thrombosis a Rare Cause of Acute Abdomen. Case Presentation

**DOI:** 10.7759/cureus.29727

**Published:** 2022-09-29

**Authors:** Mohammad Alharbi, Carlos Y Benitez, Abdullah M Alharbi, Abdulmajeed M Alharbi

**Affiliations:** 1 Surgery, Imam Mohammad Ibn Saud Islamic University, Riyadh, SAU; 2 Surgery, Hospital Royo Villanova, Zaragoza, ESP; 3 Medicine, Imam Mohammad Ibn Saud Islamic University, Riyadh, SAU

**Keywords:** anticoagulant, ct angio, abdominal pain, thrombosis, celiac artery

## Abstract

Thrombosis of the celiac artery trunk is a rare cause of acute abdominal pain which is associated with high mortality and morbidity if not diagnosed and treated early. We present an unusual case of celiac trunk artery thrombosis associated with aortic thrombosis and partial splenic infarction in a 52-year-old female who presented with acute abdominal pain. Hematological investigations failed to predict any predisposing factors. This case demonstrates the importance of considering such clinical presentation in the differential diagnosis of acute abdomen

## Introduction

Thrombosis of the celiac trunk is a rare cause of acute abdominal pain. Successful treatment depends on early diagnoses and early establishment of the blood flow by surgical or endovascular intervention. We present a 52-year-old lady who was admitted to our emergency department with acute abdominal pain and signs of peritonitis and was diagnosed with a CT scan to have non-occlusive celiac trunk thrombosis associated with aortic thrombosis splenic infarction.

## Case presentation

A 52-year-old previously healthy Saudi woman with no history of medical disease or surgical operations presented at our hospital with a sudden onset of upper abdominal pain for four days. She described the pain as a dull ache localized initially to the epigastrium, but it became severe and diffused all over the abdomen on the admission day. The pain was aggravated by food and fluid intake and relieved by hunger and was associated with frequent vomiting of bile-stained vomitus. The bowel habits were regular, and there was no blood in the stool. She was not on medication other than acetaminophen for relief of the current episode of abdominal pain. She was not on birth control pills, hormone replacement therapy, tamoxifen, or other drugs that may increase the risk of thrombosis. On clinical examination, she looked ill, pale, in pain, and not febrile Pulse was 106/minute and BP104/56. Abdominal examination revealed mild distension with generalized rebound tenderness, especially on the left upper quadrant of the abdomen. Bowel sounds were normal. The laboratory tests showed hemoglobin of 11.3 g% white blood cell 8,300/, platelet 519,000/mm3, the international normalized ratio of 1.45, and activated partial thromboplastin time of 36.30s. The liver function test, urea, creatinine and electrolytes, and serum lipase and amylase were within the normal ranges, as shown in Table 1.

**Table 1:** The laboratory results including complete blood count, urea, liver function test, international normalized ratio, and partial thromboplastin time.

Variables	Results	Normal values
Hemoglobin	11.3 g%	12-16g%
White blood cells count	8,300/mm^3^	4,000-10.500/mm^3^
Platelets	519.000/mm^3^	150-450/mm^3^
International normalized ratio (INR)	1.45	0.9-1.1
Partial thromboplastin time (PTT)	of 32.60s	10.1-12.8
Glucose random	67.9 mmol/L	4.1-7.1 mmol/L
Blood urea nitrogen (BUN)	2.1 mmol/L	3.2-8.2 mmol/L
Bilirubin	18umol/L	5-21 umol/L
Bilirubin (conjugated)	4.2	0-5
Gamma-glutamyl transferase (GGT)	12 U/L	0-38
Alkaline phosphatase	96 U/L	46-116
Alanine trasaminase (ALT)	8 U/L	7-40
Albumin	36 g/L	34-50
Total protein	67 g/L	57-82
Aspartate transaminase (AST)	15 U/L	12 -40
Serum Lipase	30 U/L	12-53
Serum Amylase	111 U/L	30-118

**Figure 1:**
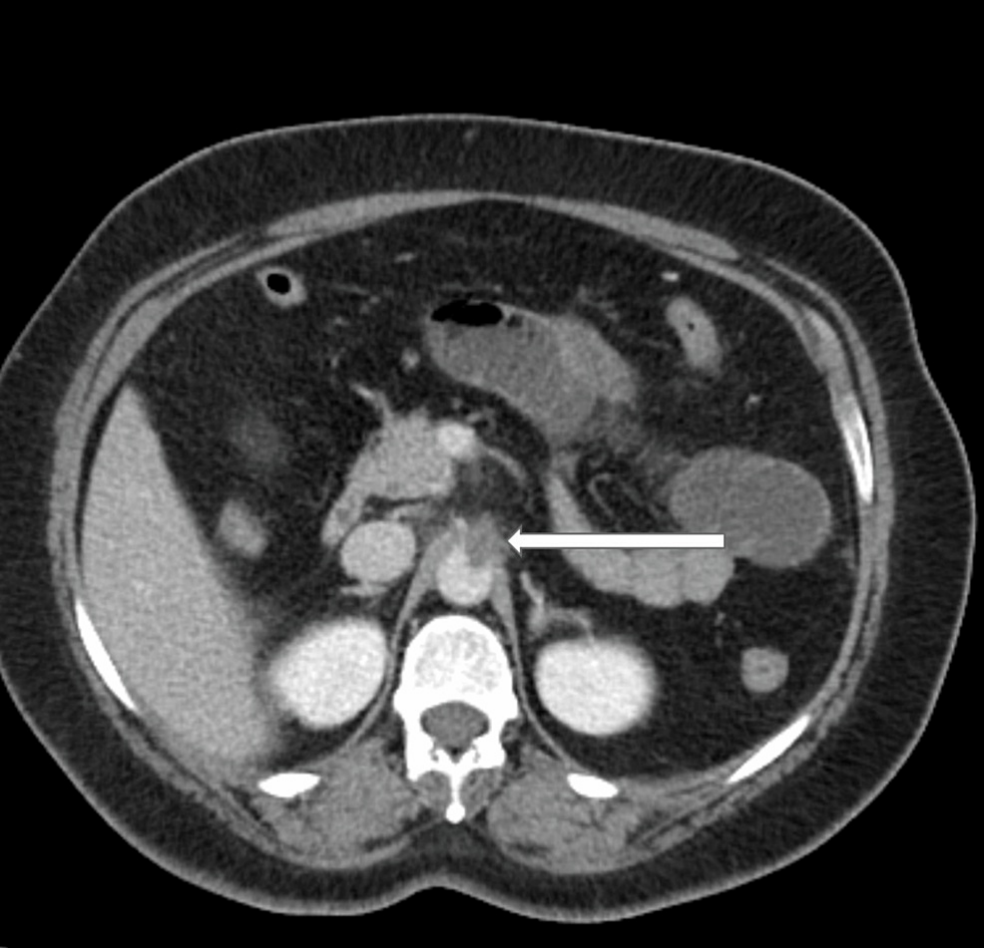
Abdominal CTA image ( axial view) showing abdominal aortic anterior mural thrombus centered on the origin of the celiac trunk artery (white arrow)

She underwent a computer tomography angiography (CTA), which showed evidence of abdominal aortic anterior mural thrombus measuring 27x11x8.5 mm in diameter (Figure 1).

**Figure 2:**
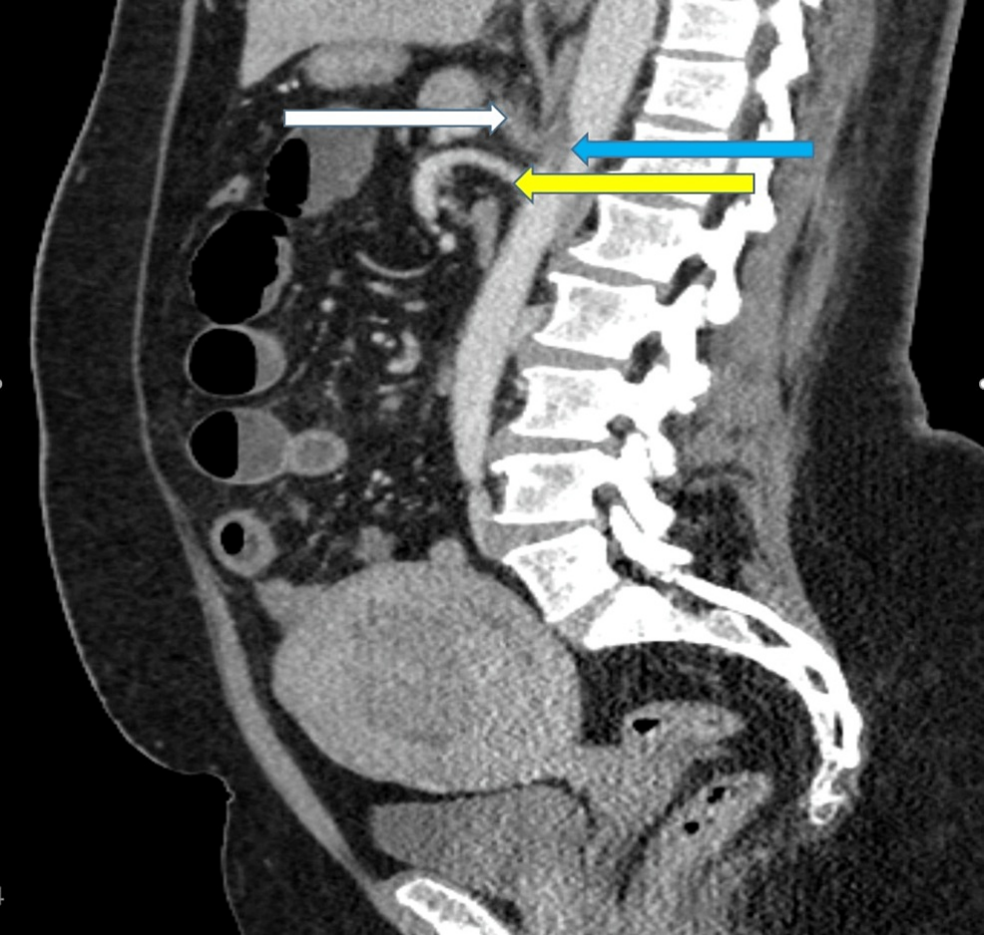
Abdominal CTA image (sagittal view) showing the aortic thrombus (blue arrow) centered on and completely occluding the origin of the celiac trunk artery (white arrow) without involving the superior mesenteric artery (yellow arrow).

**Figure 3:**
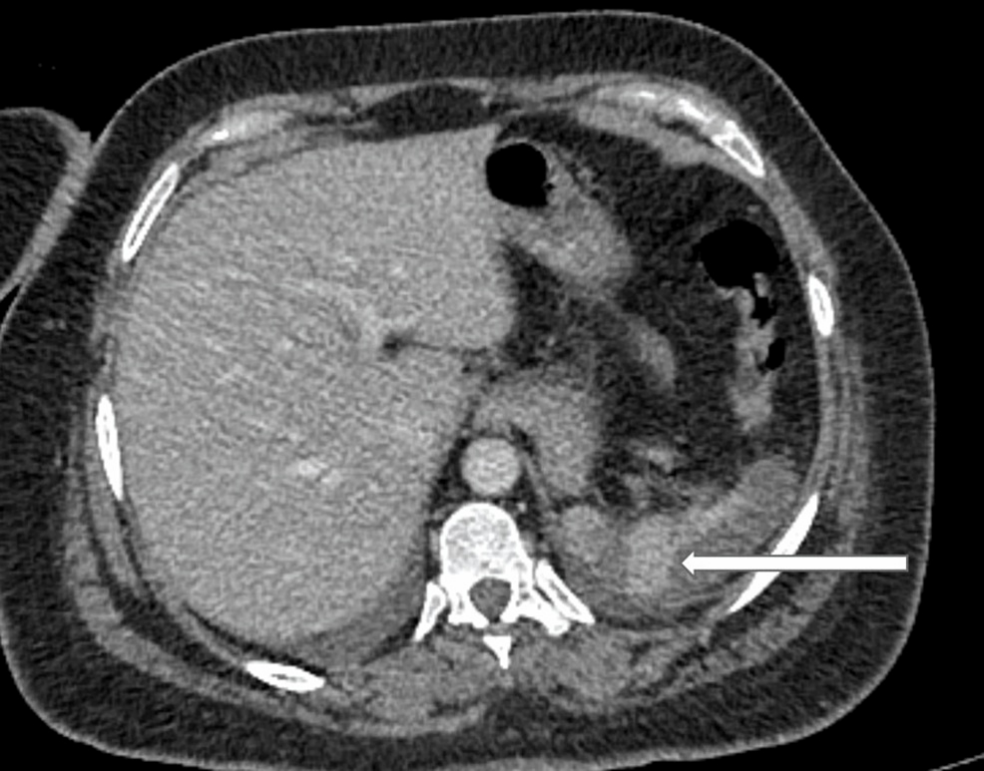
Abdominal CTA (Axial view) showing the splenic infarction (white arrow).

The aortic thrombus centered on and completely occluding the origin of the celiac trunk artery without involving the superior mesenteric artery (figure 2) with consequent near complete splenic infarction (figure 3).

**Figure 4:**
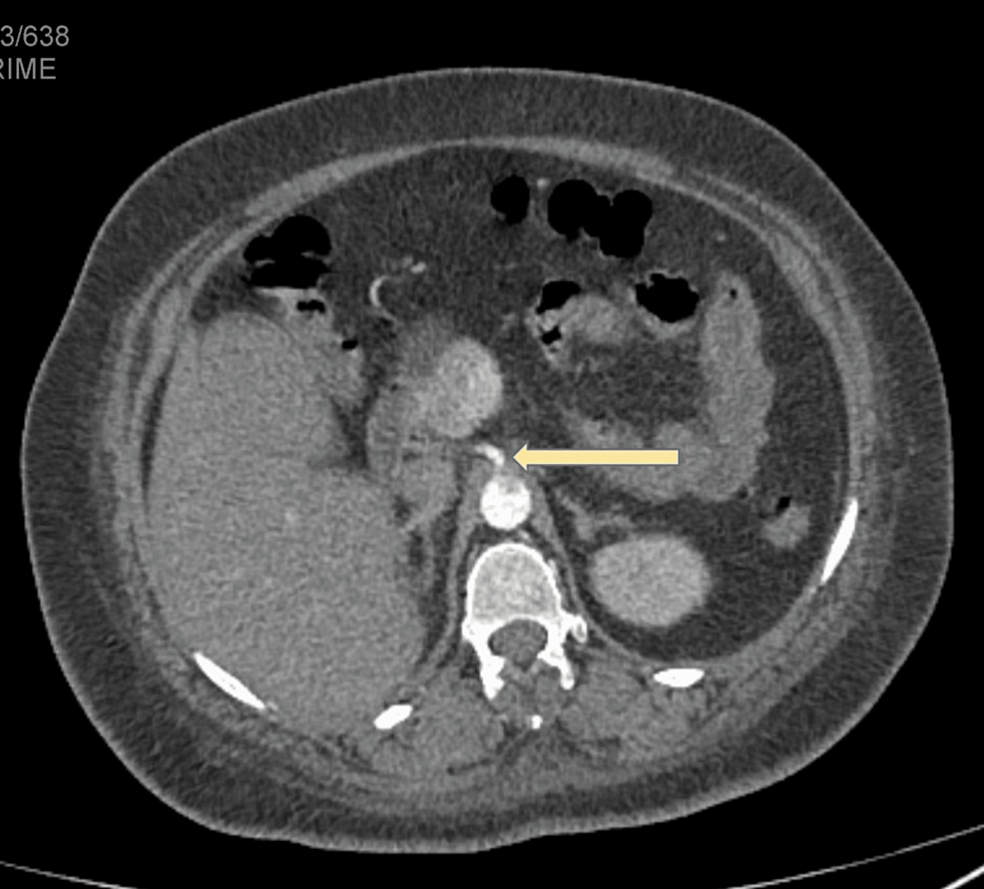
Abdominal CTA image (Axial view) showing the main hepatic artery originated independently from the aorta.

**Figure 5:**
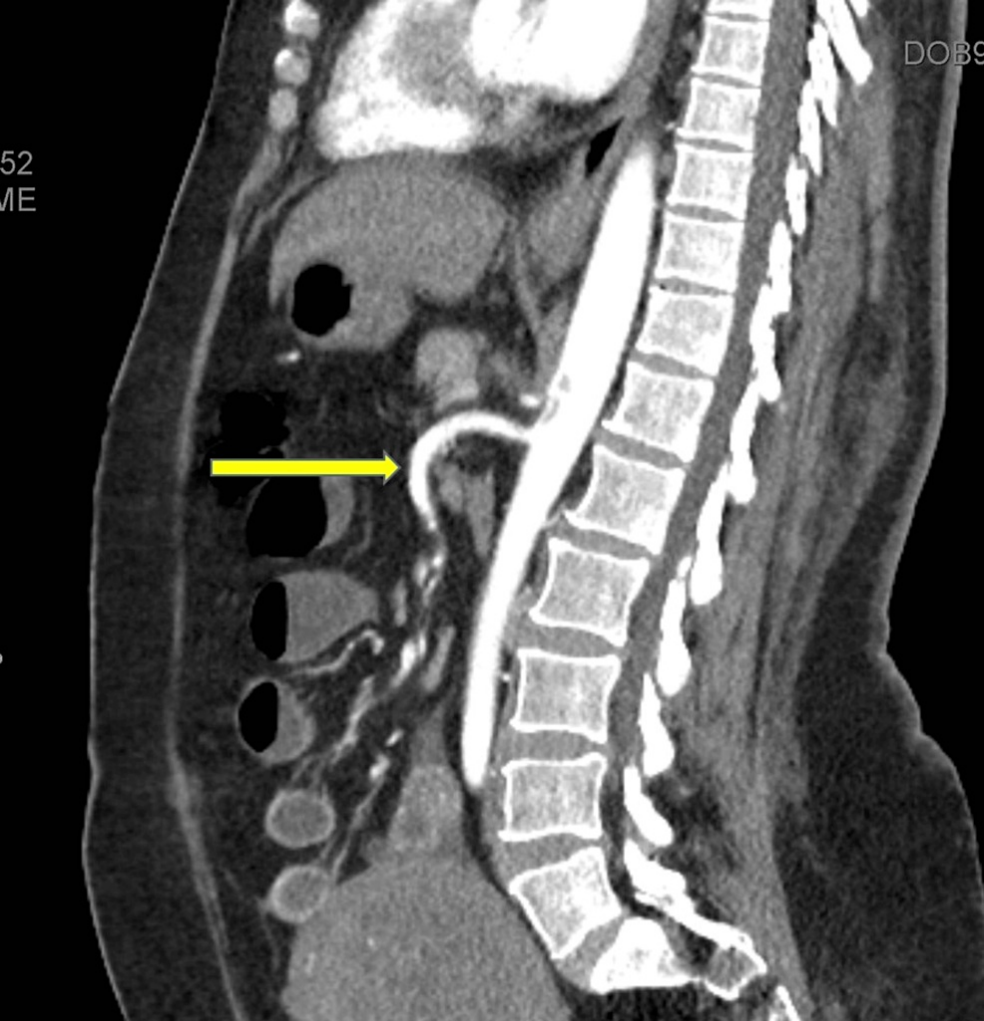
Abdominal CTAimaage (sagittal view) showing the superior mesenteric artery well opacified with the contrast media and showed no abnormalities.

The main hepatic artery originated independently from the aorta, and it was slightly narrow at its origin and was not involved with the thrombus (Figure 4). The distal two-thirds of the jejunum and proximal third of the ileum appeared distended with slight wall hypo enhancement and segmental mild wall thickening with minimal adjacent free fluid. The superior and inferior mesenteric arteries were opacified with the contrast media and showed no abnormalities (figure 5).

On the CT, the stomach and pancreas appeared normal. The liver was normal in size, shape, and parenchymal density, with no focal lesion or hepatic ischemia. Given the CTA, the patient had exploratory laparoscopy, which revealed normal bowel, stomach, and duodenum. Splenectomy was not attempted. The patient was started in full heparinization dose (5000-unit bolus and 1000 units hourly by continuous infusion). The hematologist later saw her later and was shifted to enoxaparin and aspirin. She tolerates oral feeding without having abdominal pain. She was discharged 10 days after her admission.

## Discussion

The celiac trunk, also known as the celiac artery, is a short vessel that arises from the aorta and passes below the median arcuate ligament, just as the aorta enters the abdomen at the level of the T12 vertebra. The celiac trunk is classically divided into three major branches: left gastric, common hepatic, and splenic artery. It supplies blood to the foregut, namely the distal esophagus, stomach, second part of the duodenum, liver, pancreas, gallbladder, and spleen [1].

Thrombosis of the celiac trunk is an uncommon etiology of acute mesenteric ischemia. Early diagnosis with immediate angiography and revascularization is lifesaving [2]. The disease that increases thrombotic tendency is a major etiologic factor of celiac arterial trunk thrombosis. These include atherosclerosis, Behcet disease, thrombocytosis, protein S, protein C, antithrombin III deficiency, and malignancies [3]. Other etiological disorders can be listed as acute pancreatitis, antiphospholipid syndrome, oral contraceptive drugs, diseases related to hypercoagulability, and surgical trauma [4].

Acute occlusion of the celiac trunk results in ischemic necrosis affecting mainly organs supplied by end arteries. The clinical presentations and the outcomes of celiac trunk thrombosis are variable. The stomach and spleen are more susceptible to ischemia and infarction, and dual blood supply and superior collateral flow ensure that the liver is resilient to the effects of thrombosis. Ali-Akbarian M et al. reported a case of celiac trunk thrombosis associated with a completely gangrenous stomach, the first and second portion of the duodenum, omentum, and diffuse ischemic changes and infarction of the liver and spleen [5]. In our case, the anatomical variation of the common hepatic artery arising directly from the aorta and not branching from the celiac trunk saved the liver. Gastric ischemia is uncommon as the stomach has a rich blood supply from branches of the celiac trunk and the SMA collaterals [6]. Kelekis Nl et al. reported a case of gastric fundus perforation in a patient with extensive thrombus in the celiac trunk and its branches with coexisting stenosis of the origin and proximal SMA that might have contributed to the development of irreversible gastric ischemia and necrosis [6].

Splenic infarction is common with celiac trunk thrombosis because the splenic artery is the main blood supply of the spleen. Patients may have sudden onset abdominal pain that usually resolves gradually, without clinical sequelae, and surgery is required when complications arise [7]. An uncomplicated spleen infarction can be safely managed with medical treatment, but early surgery is needed when complications of the infarct, including abscess and rupture, have occurred [7]. Successful treatment depends on early diagnosis, effective surgical or endovascular intervention to re-establish blood flow, resection of necrotic parts, and good intensive care unit management [3]. Our case was diagnosed early, and the anticoagulant treatment began at an early stage, so the outcome was successful.

## Conclusions

Thrombosis of the celiac trunk is a rare cause of acute abdominal pain. The clinical presentations and the outcomes of celiac trunk thrombosis are variable. Diagnosis depends on a high index of suspicion, and CTA is the gold standard to confirm the diagnosis. Successful treatment depends on the early establishment of the blood flow by anticoagulant, surgery, or endovascular intervention.
